# Impact of COVID-19 on maternal healthcare in Africa and the way forward

**DOI:** 10.1186/s13690-021-00746-6

**Published:** 2021-12-10

**Authors:** Edward Kwabena Ameyaw, Bright Opoku Ahinkorah, Abdul-Aziz Seidu, Carolyne Njue

**Affiliations:** 1 School of Public Health, Faculty of Health, University of Technology Sydney, Sydney, NSW 2007 Australia; 2 Department of Estate Management, Takoradi Technical University, P.O.Box 256, Takoradi, Ghana; 3 Centre for Gender and Advocacy, Takoradi Technical University, P.O.Box 256, Takoradi, Ghana; 4 College of Public Health, Medical and Veterinary Sciences, James Cook University, Townsville, Queensland Australia

**Keywords:** COVID-19, Maternal healthcare, Africa, Reproductive health, Public health

## Abstract

**Background:**

The impact of COVID-19 is weighing heavily on many African countries. As of November 14th 2021, 6,109,722 cases had been recorded with 151,173 deaths and 2.5% case fatality rate. Studies reveal substantial morbidity and socioeconomic impacts when accessing quality maternal healthcare including fear of infection and the containment measures in place, including social distancing and community containment. The pandemic has put additional strain on healthcare systems that are overburdened and under-resourced even in normal times and has exposed the vulnerabilities of high-risk population groups in addressing critical healthcare concerns. This study presents a mini review of how COVID-19 has disrupted maternal healthcare in Africa, and it further proposes ways to improve the situation.

**Main body:**

COVID-19 has disrupted antenatal, skilled birth, and postnatal family planning services. Women and girls are vulnerable to the impact of COVID-19 on several fronts and represent a group whose needs including antenatal, skilled birth, and postnatal family planning services have been disrupted, leading to unmet needs for contraception and an increase in unintended pregnancies. Restricted travel due to the fear and anxiety associated with contracting COVID-19 has resulted in delays in accessing prompt skilled care and essential healthcare services such as pregnancy care, immunisation, and nutritional supplementation. Misconceptions relating to COVID-19 have prompted concerns and created distrust in the safety of the healthcare system. Innovative measures are required to address these obstacles and ensure women are not denied access to available, accessible, acceptable, and quality maternal healthcare services in spite of COVID-19.

**Conclusions:**

In the immediate term while physical distancing measures remain in force, deliberate effort must be made to provide evidence-based guidelines, good practice and expert advice that addresses the unique sexual and reproductive health context of African countries. Efforts to train and motivate healthcare providers to adopt online, remote approaches such as use of telemedicine, and expand the involvement of frontline maternal healthcare providers to deliver information on the availability of services through phone-based referral networks, culturally appropriate social media, community radio and folklore messaging strategies are critical to mobilise and secure community confidence in the safety of sexual and reproductive health and maternal care services.

## Background

Although the novel coronavirus disease 2019 (COVID-19) has affected all continents, its impact varies and has been tied to the resilience levels of health systems [1]. In the case of Africa, by November 14th 2021, 6,109,722 cases had been recorded, with 151,173 deaths and 2.5% case fatality rate [2]. Reproductive healthcare is one of the core aspects that has been severely threatened [3]. This is critical considering the limited utilisation of essential maternal health services due to pre-existing systemic barriers faced by African women in their quest to access antenatal care (ANC) [4], facility-based childbirth [5] and family planning services [6]. For instance, Adedokun and Yaya using Demographic and Health Survey (DHS) data from 31 African countries, noted that 13% of the women did not utilise ANC at all, whilst 35% utilised ANC services partially [7]. Another study of 28 African countries based on DHS data revealed that facility-based childbirth ranges from 23% with an average of 66% [8]. In terms of prevalence and maternal health impact of COVID-19 in Africa, the worst affected countries include South Africa, where a 3.4% rise in perinatal mortality and 5% decline in family planning services has occurred due to COVID-19 [9]. Others are Liberia, Uganda, Zambia, and the Democratic Republic of Congo [10]. In spite of these, COVID-19 measures were instituted in a number of African countries such as South Africa [11], Kenya [12], Ghana [13] and Africa at large [14, 15] have extensively focused on how to cushion the citizens from the downturns in earnings and other similar measures to revamp the economy. Workable interventions to sustain and potentially even improve the ailing reproductive health system have been largely omitted in COVID-19 measures in Africa [16, 17]. This affirms the prediction by the United Nations Population Fund (UNFPA) that during crises, reproductive health needs are likely to be overlooked [17].

Since Africa bears the greatest proportion of global maternal and newborn mortality, unintended pregnancies and unmet need for contraception [18, 19], more attention is required to determine the best way to offer these essential services, otherwise these maternal issues may be exacerbated further during the pandemic. This mini review, therefore, takes stock of the extent to which ANC, skilled birth and family planning services have been disrupted by COVID-19 in Africa. The review further shares innovative measures required to lessen the impact of COVID-19 on these indispensable maternal health services.

## Main text

### How has COVID-19 impacted ANC services?

ANC has proven to be one of the most effective means of timely disease detection and treatment strategies during pregnancy. During ANC, pregnant women are introduced to the use of iron and folate supplements to control and treat anaemia, intermittent preventive treatment for malaria as well as immunisation against tetanus and tuberculosis, and detection of sexually transmitted infections (STIs) including HIV and AIDS to prevent mother to child transmission [20]. In the wake of the COVID-19 pandemic, most of these essential health services for pregnant women have been delayed or shifted, while other women do not seek ANC services at all as found in Ethiopia [21], Kenya [22], Ghana [23], Burkina Faso [24], Uganda [25], Nigeria [26] and several other African countries [24, 27]. Pregnant women who become infected with COVID-19 are also likely to suffer from hypercoagulability [28, 29], making ANC even more crucial for them.

In most cases, pregnancy is associated with a hypercoagulable state as infected women may be more susceptible to venous thromboembolism partly due to a decrease in exercise [30, 31]. Based on these, providers of ANC should be meticulous while assessing their patients. Dashraath et al. [30] advocate that foetal growth restriction could be observed due to maternal hypoxia and that this highlights the need for at least one ultrasound foetal growth assessment post maternal COVID-19 recovery. Dashraath and colleagues further suggested that foetal kick counts can be utilised in place of non-stress tests. Blood pressure could also be measured at home [30], while urine and blood tests could be conducted at nearby facilities whenever possible and reported to the treating obstetrician by phone [31].

### Effect of COVID-19 on skilled birth attendance in Africa

According to UNICEF [32], the safest place for childbirth is a well-functioning health facility with the support of a skilled birth attendant. However, amid a global crisis such as COVID-19, many women may end up giving birth at home without appropriate support, particularly among those in rural parts of the continent [33]. This is more critical considering the pre-existing low skilled birth attendance in Africa relative to other regions [34]. Learning from the experience of the Ebola virus in Africa, a study of the virus estimated that during the outbreak, facility delivery with skilled birth attendance reduced by 8% points [35]. Possible reasons for the reductions were that there were fears of contracting the Ebola virus at health facilities, distrust of the health system and rumours about the source of the disease [35].

Similar reports have emerged across the African continent within this era of COVID-19. For instance, in Burundi, childbirth with skilled birth attendants reduced from 30,826 to 4,749 (i.e. 85%) in April 2020 compared with April 2019 [36]. In Ghana, a pregnant woman died at a hospital after healthcare providers abandoned her due to fear of contracting COVID-19 [37]. Moreover, substantial variation exists with respect to challenges faced by women in accessing skilled birth across Africa. In the case of South Africa [38] and Senegal [39], the disparity between the rich and the poor is phenomenal, while, for some other countries such as Ghana [40], Nigeria [41] and Tanzania [42], transportation challenges dominate.

### COVID-19 and postnatal care services, including family planning in Africa

The post-partum period is one of the most crucial times in the lives of women and newborns [43, 44]. This is the period where a woman’s need for family planning services is greatest as well as child welfare services including immunisation against childhood killer diseases [45]. The World Health Organisation (WHO) indicates that more than 90% of post-partum women want to delay or avoid another pregnancy, yet two-thirds are not using contraception. Evidence suggests that an earlier outbreak of Ebola led to a drop in the delivery of postnatal care services including family planning in Sierra Leone and Liberia [46]. Another major impact is the stock-outs of contraceptives in terms of supply. The UNFPA has indicated that due to COVID-19, about 46 countries that usually receive contraceptive support will experience a decline in the supply of major contraceptives such as implants, IUDs, condoms and oral contraceptives pills [47]. The Guttmacher Institute has also stated that if the current situation persists, there will be around a 10% decline in contraceptive usage due to stock-outs, unavailable providers and closed clinics. This will also contribute to about 48 million women having an unmet need for contraception, leading to about 15.4 million unintended pregnancies, which could further lead to 1.7 million obstetric complications [48]. This will work against the efforts made to improve maternal healthcare, especially in the fight against maternal mortality [49]. The African continent would eventually bear the greatest social and economic burden, as about one in five women on the continent have an unmet need for family planning [50].

### The way forward

The African countries require context-appropriate and unique approaches to augment sexual and reproductive healthcare during the COVID-19 crisis. Governments of African countries that may plan a lockdown in the case of a second wave should create routine public awareness about the importance of maternal health services. This may include strengthening media coverage to motivate mothers to access sexual and reproductive health services with all precautionary measures in place. This can be complemented by the promotion of online antenatal, postnatal, and family planning services. For instance, group/individual sessions via online platforms, static web resources, emails, support groups, telehealth or mobile phone appointments may be useful. To meet the needs of both literate and illiterate women, community-based information centres can also be used as a mode of education.

Additionally, efforts should be made to train and motivate community health volunteers to encourage and equip them to perform home visits, provide counselling and identify mothers who require specialised care. This could be enhanced through streamlined referral to the next level of healthcare and practical guidelines, stringent supervision and monitoring to ensure the continuity of such efforts.

Governments of African countries need to reinforce their commitments towards achieving herd immunity for their citizenry by stepping up safe and effective vaccine procurement efforts. Thus, there is the need to secure adequate quantities of COVID-19 vaccines through COVAX and strengthen COVID-19 vaccine campaigns/education to enhance the people’s confidence in vaccination campaigns and eschew fake news in circulation about the vaccine.

Health facilities should also ensure that core sexual and reproductive health services such as antenatal, delivery, postnatal and family planning services are available and accessible to all women throughout the COVID-19 season. However, these services should be delivered in strict adherence to all COVID-19 protocols sanctioned by the WHO, including social distancing and wearing of nose masks [51]. Further, a limited number of women could be scheduled for maternal health services at a specified time to reduce the chances of overcrowding at the health facilities. Cost of testing could be made free across African countries where governments can absorb, not only in public health facilities but in the private sector, to increase uptake and early treatment for women. As a number of African countries are struggling to get adequate COVID-19 vaccines [52], there is the need to ensure that pregnant women, as a priority population, are early recipients during the vaccines’ rollout and distribution.

In strengthening the resilience of maternal and child health service delivery, unwavering political commitment is required from African governments. Specifically, there is the need to disaggregate maternal healthcare challenges exacerbated by COVID-19 from the potential new challenges resulting from the pandemic and advance feasible strategies to tackle them whilst prioritising the country’s capacity and contextual circumstances.

A preliminary analysis by the WHO in 28 African countries indicates that several African countries are instituting measures to assuage the impact of COVID-19 on reproductive health [53]. These measures comprise the provision of mobile family planning services, reorganizing antenatal care services and provision of personal protective equipment to minimize COVID infection, self-care options for oral and injectable contraceptives, as well as public-private partnerships to offer contraceptives and other essential family planning commodities. These countries include Cote d’Ivoire, the Democratic Republic of the Congo, Seychelles, Eswatini, Mali, South Africa and Senegal [53]. With these measures, it is anticipated that the current and potential impacts of COVID-19 on maternal health will decline to the barest minimum.

## Conclusions

This mini review has reflected on how COVID-19 has affected three reproductive health services: antenatal care, skilled birth and postnatal care in Africa. The review shows that COVID-19 has immensely affected reproductive health services adversely in Africa since its penetration of the continent.

A united front is required from governments and all stakeholders in Africa to institute and advocate fit-for-purpose measures to save maternal and newborn lives. Some key strategies worth instituting include encouraging healthcare providers to adopt innovative and technological approaches to administer all services that can be offered remotely, promote the standard of care improvements and contribute to the co-design of public health messaging to dispel fears in communities. Further methods include working to mitigate risk factors to avoid disrupted access to maternal healthcare.

Considering that some healthcare providers have been able to abandon pregnant women to the point of death, much more sensitisation is required to inspire compassion, empathy and professionalism in frontline maternal healthcare providers so that they can offer professional and humanised services amid the COVID-19 pandemic. Although countries across the continent share some similarities, it is worth mentioning that specific contextual and country-level characteristics of women seeking reproductive health services must be prioritised to have fit for purpose and contextually relevant reproductive health care models for the varied populace.

## Data Availability

Not applicable.

## Abbreviations

AIDS: Acquired Immunodeficiency syndrome
ANC: Antenatal care
COVID-19: Coronavirus 2019
HIV: Human Immuno-deficiency virus
IUD: Intrauterine device
SRH: Sexual and reproductive health
UNFPA: United Nations Population Fund
UNICEF: United Nations Children’s Fund
WHO: World Health Organisation
